# Expression patterns of *Brassica napus* genes implicate *IPT, CKX*, sucrose transporter, cell wall invertase, and amino acid permease gene family members in leaf, flower, silique, and seed development

**DOI:** 10.1093/jxb/erv133

**Published:** 2015-04-04

**Authors:** Jiancheng Song, Lijun Jiang, Paula Elizabeth Jameson

**Affiliations:** ^1^School of Life Sciences, Yantai University, Yantai 264005, China; ^2^School of Biological Sciences, University of Canterbury, Christchurch 8140, New Zealand

**Keywords:** Cell division, cytokinin, cytokinin oxidase/dehydrogenase, food security, forage, homoeologues, isopentenyl transferase, seed, sink, source, transcriptome, yield.

## Abstract

Forage brassica (*Brassica napus* cv. Greenland) is bred for vegetative growth and biomass production, while its seed yield remains to be improved for seed producers without affecting forage yield and quality. Cytokinins affect seed yield by influencing flower, silique and seed number, and seed size. To identify specific cytokinin gene family members as targets for breeding, as well as genes associated with yield and/or quality, a *B. napus* transcriptome was obtained from a mixed sample including leaves, flower buds and siliques of various stages. Gene families for cytokinin biosynthesis (*BnIPT1, 2, 3, 5, 7, 8* and *9*), cytokinin degradation (*BnCKX1* to *BnCKX7*), cell wall invertase (*BnCWINV1* to *BnCWINV6*), sugar transporter (*BnSUT1* to *BnSUT6*) and amino acid permease (*BnAAP1* to *BnAAP8*) were identified. As *B. napus* is tetraploid, homoeologues of each gene family member were sought. Using multiple alignments and phylogenetic analysis, the parental genomes of the two *B. napus* homoeologues could be differentiated. RT-qPCR was then used to determine the expression of gene family members and their homoeologues in leaves, flowers, siliques and seeds of different developmental stages. The expression analysis showed both temporal and organ-specific expression profiles among members of these multi-gene families. Several pairs of homoeologues showed differential expression, both in terms of level of expression and differences in temporal or organ-specificity. *BnCKX2* and *4* were identified as targets for TILLING, EcoTILLING and MAS.

## Highlight

Increases in seed yield must occur without impacting seed or plant quality. Specific *CKX* gene family members were identified as targets for breeders along with transporter and metabolic genes as quality markers.

## Introduction

Food security is reliant on security of seed production. Seed of many different species will need to be produced in increasing amounts to meet the demands for direct human and animal consumption, for the production of forage for animals, and also for the production of vegetables. A step change is needed in plant breeding to meet the demand for seed under a changing environment of reduced inputs (water, fertilizer, herbicides and pesticides), and a changing global climate.

The Brassicaceae represents the most diverse family used as a vegetable for human consumption and as forage for animal grazing, and consistency of yield and quality of seed are traits not yet optimized by the *Brassica* seed industry in New Zealand (Hampton *et al.*, 2012). In this research, we have focused on *Brassica napus* cv. Greenland, a forage brassica used for winter grazing of cattle in New Zealand. Forage brassica is purposely bred for optimized vegetative growth and biomass production, while its seed yield remains to be improved for seed producers without affecting forage yield and quality.

The cytokinins have been implicated as a limiting factor in the establishment of sink number and sink size in legumes, cereals and *Arabidopsis*. Ectopic expression of an *IPT* gene has been shown to increase seed yield in a variety of plants (see review by Guo and Gan, 2014). Notwithstanding the implication that cytokinin is limiting yield, high levels of endogenous cytokinins are routinely detected in developing fruits and seeds. However, the peak of cytokinin is often transitory. For instance, the amounts of endogenous cytokinin in developing cereal grains have been shown to change rapidly (Morris *et al.*, 1993). In wheat, the endogenous changes are compressed into just a few days after anthesis (Jameson *et al.*, 1982), at the time when sink size is established during the phase of free nuclear divisions and cellularization of the endosperm (Bennett *et al.*, 1973). The rapid changes in endogenous levels of cytokinins in wheat have been linked to the expression of specific members of the cytokinin biosynthesis (*IPT*), degradation (*CKX*), *O*-glucosylation and ß-glucosidase gene families (Song *et al.*, 2012).

Similarly in maize, Brugière *et al.* (2008) suggested that the coincidence of the expression of *ZmIPT2,* activity of its enzyme, peak endogenous cytokinin levels and the phase of cell division is indicative of cytokinin as a necessary component of endosperm cell division. However, they also showed, using immunolocalization, ZmIPT protein not only in the endosperm/embryo during the cell division phase, but also located in the endosperm transfer cell layer both during the phase of cell division and later in seed development.

In legumes, cytokinin is limiting even to pod set. In lupin, where the majority of flowers will abort, application of cytokinin prevented this abortion (Aitkins and Pigeaire, 1993). Emery *et al.* (2000) correlated cytokinin form with flower/ovary abortion, and increased pod set in plants by ectopically expressing an *IPT* gene (Atkins *et al.*, 2011). Moreover, while xylem-supplied cytokinin does reach the pod wall of lupins (Jameson *et al.*, 1987), little of the translocated cytokinin crossed the apoplastic space between the seed coat and embryo (Singh *et al.*, 1988; Letham, 1994), whereas adenosine did (Noodén and Letham, 1984). Detailed GC-MS data on developing white lupin showed a high, transient peak of cytokinin in the liquid endosperm of developing seeds (Emery *et al.*, 2000). The conclusions from work on legumes have been that maternally supplied cytokinin via xylem and/or phloem is limiting to pod and seed set (Emery *et al.*, 2000), but that the developing embryo is dependent on cytokinin biosynthesis in the filial tissues (Singh *et al.*, 1988; Emery *et al.*, 2000). In *Arabidopsis*, Day *et al.* (2008) showed elevated levels of core cell cycle genes and genes involved in cytokinin biosynthesis (*IPT8*) and signalling in the syncytial endosperm, and suggested that the chalazal endosperm was directing syncytial endosperm development via cytokinin signalling (Day *et al.*, 2008).

The seminal work of Ashikari *et al.* (2005) on yield of rice has shown the possibility that the cytokinins could be a direct target for plant breeders. They showed that a QTL for increased grain number was associated with the gene for cytokinin oxidase/dehydrogenase (*CKX*), which codes for the enzyme that inactivates cytokinin. Indeed, Ashikari *et al.* (2005) showed that the rice cultivars with increased seed number had mutated forms of a specific *OsCKX* gene family member—*OsCKX2*. Seed number was increased in lines in which a mutation for reduced activity or a null mutation in *OsCKX2* had occurred (Ashikari *et al.*, 2005). Additionally, down-regulation of *HvCKX1* or *HvCKX9* by RNA interference in barley led to both increased seed number and seed size (Zalewski *et al.*, 2010, 2012, 2014). Moreover, the significant yield increase caused by the down-regulation of *HvCKX1,* which was shown to be the more highly expressed gene family member during kernel development, was inherited across four generations (Zalewski *et al.*, 2014). Recently, Zhang *et al.* (2012) suggested that variants of *TaCKX6*-D1, a wheat orthologue of rice *OsCKX2*, were significantly associated with grain weight but not grain number. In Eudicots, a double *ckx* mutant of *Arabidopsis* showed a 55% increase in yield (Bartrina *et al.*, 2011) leading to the suggestion that the role of *CKX* genes in determining seed yield has been evolutionarily conserved and is of functional significance for all or most flowering plants (Bartrina *et al.*, 2011).

The original work of Mothes and Engelbrecht (1963), in which cytokinin applied to a leaf was shown to attract nutrients to the site of application, established a role for cytokinin in enhancing sink activity independent of a role in cell division. Subsequently, cross-talk between cytokinin and genes involved in sink activity was shown by Ehneß and Roitsch (1997) using cultured cells *in vitro,* where cytokinin enhanced the simultaneous expression of a cell wall invertase (CWINV) (which irreversibly catalyses the breakdown of sucrose to fructose and glucose) and a hexose transporter gene in *Chenopodium rubrum* (Amaranthaceae). Subsequently, the induction of CWINV as an essential component of cytokinin-induced delay of senescence was also shown (Balibrea *et al.*, 2004) with Hwang *et al.* (2012) suggesting that cytokinin-mediated senescence delay is caused by an increased sink activity via the direct activation of CWINV activity.

Significantly, a gene coding for a CWINV has been shown to be necessary for normal seed development. Research has shown that the maize cell wall invertase-deficient mutant, *miniature1* (*mn1*), has impaired development of the endosperm and pedicel cells (Cheng *et al.*, 1996; Rijavic *et al.*, 2009) and that seeds of the mutant have altered hormone levels (LeClere *et al.*, 2010; Rijavic *et al.*, 2009). Rijavic *et al.* (2009) suggested that the effect of cytokinin on seed size is both direct, through an effect on cell cycle-related genes, and indirect, through the action of sugar signalling. They also suggested that cross-talk between cytokinin and CWINV might enhance phloem unloading and sugar import into the maize endosperm (Rijavic *et al.*, 2009).

Wang and Ruan (2012) showed that *CWINV* in both cotton seeds and *Arabidopsis* seeds was more abundant in the chalazal endosperm undergoing endoreduplication but much weaker in cellularizing endosperm. They suggest that CWINV may be promoting nuclear division probably through sugar signalling and that, by cross-talk between hormones, invertase-mediated sugar signalling may regulate the expression of cell cycle control genes (Wang and Ruan, 2012). Both sugars and cytokinin have been directly implicated in control of the plant cell cycle. Sugar availability has been shown to play a major role during the G1 phase of the cell cycle by controlling the expression of D-type cyclins. Both *AtCYCD2* and *D3* responded to sucrose availability (Riou-Khamlichi *et al.*, 1999, 2000). While *CYCD2* expression was independent of hormones, a continuing response of *CYCD3* required both sucrose and cytokinin. Both the sucrose and the cytokinin responses were direct and did not require protein synthesis (Riou-Khamlichi *et al.*, 2000).

If cytokinin levels are increased in seeds of forage brassica by selecting for reduced activity of CKX, a concomitant increase in CWINV activity might be anticipated. However, if seed yield is to be increased and quality maintained in both seed and forage, then a co-ordinated increase in activity of transporters is also required to supply the carbon and nitrogen backbones for growth and metabolism, and eventually storage. Assimilate translocation from source to sink tissues depends on transporter genes including sucrose transporters (SUTs). Indeed, Li *et al.* (2011) suggested that assimilate translocation is the most critical limiting factor for seed yield in *Brassica*, and showed that *BnSUT1* was associated with a QTL for yield in rapeseed (Li *et al.*, 2007).

In addition to a supply of carbon, developing pods and seeds have competitive requirements for nitrogen, initially for metabolic enzymes in the expanding pod and developing seeds and subsequently for the formation of seed storage proteins. Tegeder (2014) suggests that sink development and sink nitrogen levels depend on the amounts of nitrogen (and carbon) that are transported in the phloem. Transport of nitrogen from the leaves to sink tissues is in the form of amino acids in non-leguminous plants and involves the activity of amino acid permeases (AAPs) (Tegeder, 2014).

In this work we had two key aims. The first was to identify *CKX* gene family members expressing specifically in developing siliques and seeds, as a preliminary to identifying mutant *CKX* forms for breeding. From a breeding perspective utilizing TILLING and EcoTILLING strategies, detection of loss of function mutants of *CKX* should be easier than detection of mutants with increased expression of *IPT*. The second aim was to obtain an overall picture of the expression of *IPT, CKX, SUT, INV* and *AAP g*ene family members during development of forage brassica plants. A *B. napus* transcriptome was obtained from a mixed sample of plant material including leaves, flower buds and siliques of various stages. This was mined for the gene families of interest. Subsequently, we determined, using RT-qPCR, the expression of the *BnIPT* and *BnCKX* gene family members as well as of genes likely to be involved in source-sink relationships during leaf, flower and silique development in forage brassica. As *B. napus* is tetraploid, homoeologues of each gene family member were sought.

## Materials and methods

### Plant material

Forage brassica, *Brassica napus* cv. Greenland, was sourced from a commercial seed field in Tinwald, South Canterbury, New Zealand (December 2010). Tissues at three stages of leaf and flower and seven stages of silique development were collected from six plants at similar developmental and physiological status, and were immediately placed into liquid nitrogen and then stored at −80°C.

Leaf 1 were identified as being small, very young leaves near the top of the extending plant. Leaf 2 were larger, fully expanded functional leaves nearer to the middle of the plant. Leaf 3 were senescing leaves nearest the base of the plant. Flower 1 were young, closed flower buds of 3–4mm in length, with no yellow petals visible. Flower 2 were still just closed flower buds of 6–8mm in length, with the yellow petals tips visible. Flower 3 were newly opened flowers ready to be pollinated. The seven stages of silique were taken from two days after pollination (DAP) through to fully expanded siliques, with lengths of P1=15–20mm, P2=25–30mm, P3=35–45mm, P4=50–55mm, P5=60–65mm, P6=70–75mm, and P7=70–80mm.

### RNA isolation and cDNA synthesis

Total RNA was extracted from up to 100mg of frozen samples using TRIzol Reagent (Invitrogen, Carlsbad, CA, USA) following the manufacturer’s instructions and immediately stored at 20°C. The integrity and quality of isolated RNA was assessed by running 1 μl samples on a 1% (w/v) agarose gel. The concentration and purity of the total RNA was assessed using a Nanodrop™ spectrophotometer.

Extracted RNA was converted to cDNA through reverse transcription. Approximately 1 µg of total RNA, 50U Expand Reverse Transcriptase (Roche, Mannheim, Germany), 50 pmol oligo (dT) primers and 100 pmol random hexamer (pdN6) primers were used in a 20 μl reaction. The final reaction mix was incubated at 42°C for 2h, and then at 70°C for 15min to deactivate the enzyme. The cDNA was diluted 10-fold with nanopure water and stored at −20°C.

### Gene isolation and sequence analysis

Sequences of family members from candidate genes of interest and their homoeologues were isolated from RNA-Seq transcriptome data. An RNA pool of combined RNA samples extracted separately from multiple developmental stages of leaves, flowers and siliques was used to construct the cDNA library, which was then sequenced using an Illumina HiSeq2000 genome analyzer at the Beijing Genomic Institute (BGI) customer service.

Orthologue sequences of *IPT*, *CKX*, *SUT*, *CWINV* and *AAP* from *Arabidopsis* and *Brassica* species available in the GenBank database were used as query sequences to BLAST search our *B. napus* transcriptome data using prfectBLAST 2.0. The putative sequences were verified via BLAST searching the GenBank database and multiple sequence alignment with representative orthologue sequences in closely related species.

The newly identified sequences and their orthologues in *Arabidopsis* and other *Brassica* species were used to construct Neighbor-Joining (NJ) phylogenetic trees using ClustalX software with 1000 bootstrap replicates. Each tree was rooted with an out group orthologue sequence. The GenBank accession numbers for the nucleotide sequences are listed in Supplementary Table S1.

### Quantitative reverse transcription polymerase chain reaction

Quantitative RT-PCR was used to measure relative gene expression of the individual family members across the various plant tissues as they developed. Specific PCR primers were designed for each family member of the five genes of interest and their homoeologues within the subgenomes A and C using Primer Premier 6.0. In most cases, four primer pairs were designed and the best one was chosen for gene expression analysis after PCR testing (Supplementary Table S2). A volume of 20 µl was used for all qPCR reactions in a Rotor-Gene Q (Qiagen) real-time PCR instrument, using home-made SYBR Green master mix or a KAPA SYBR® FAST qPCR Kit (Kapa Biosystems, Boston, USA). PCR products for each target sequence were Sanger sequenced to confirm homology to genes already identified in various gene databases (e.g. NCBI). PCR systems were then optimized and the amplification efficiency determined. Two reference genes, elongation factor gene EF and GAPDH, were used as internal controls. For each cDNA sample, the Ct values of each target gene were corrected using a correction factor (CF) calculated as described previously (Song *et al.*, 2012). Three technical replicates for each of two biological replicates were carried out for each sample set. The expression values relative to EF and GAPDH were calculated based on the methods of Pfaffl (2001) and modified as described in Song *et al.* (2012). Data for the second biological replicate are shown in Supplementary Table S3.

## Results

### Silique and seed development

**Fig. 1.**
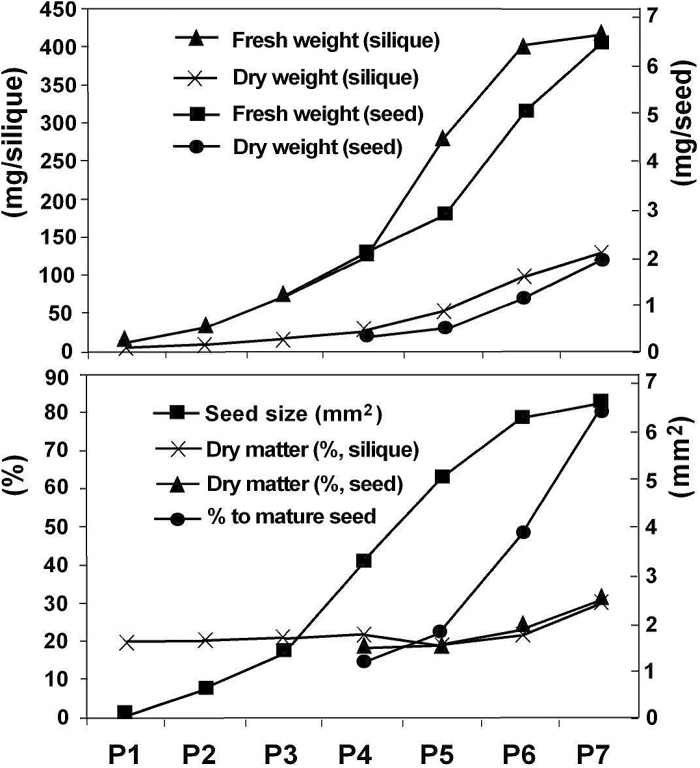
Description of developmental stages of *B. napus* silique and seed samples used for gene expression analysis. Silique length (mm): P1, 15–20; P2, 25–30; P3, 35–45; P4, 50–55; P5, 60–65; P6, 70–75; P7, 70–80. Embryo developmental stages: P4, heart to torpedo; P5, linear to curled cotyledons; P6, green cotyledons.

Siliques were collected at different stages of maturity (based on length), from secondary or tertiary branches with the most mature siliques collected from the base and the least developed from the upper branches of indeterminate forage brassica plants. Siliques were separated into size classes with the largest included in P6 and P7 samples. Simultaneous with silique elongation, seed size was also increasing to a maximum in P6 and P7 siliques (Fig. 1). Immature seeds observed in P1–P3 siliques were mostly seed coat and liquid endosperm. Seeds from P1 and P2 siliques were transparent, seeds from P3 siliques were yellowish, those from P4 slightly green, and those from P5–P7 were bright green. Embryo dissection showed that the embryos were at heart to torpedo stages in P4 siliques. Early linear cotyledon stage to curled cotyledon stage embryos were found in P5 siliques, and well formed green cotyledons occupied the whole of the seed from P6 siliques. Seeds from P4–P7 were able to be dissected from the siliques for gene expression analysis.

Fresh weight and dry weight of siliques increased noticeably from P4. Dry weight of the seeds was assessed against a commercial seed lot: seeds removed from P6 siliques had accumulated approximately 50% of final dry matter, and in P7 siliques up to 80%. While the rate of fresh weight increase of siliques decreased between P6 and P7, the fresh weight of seeds in P7 siliques was still increasing, indicating that the seeds had not reached the stage of desiccation (Fig. 1).

### Transcriptome analysis

The RNA-Seq from a cDNA library constructed using pooled RNA samples from leaves, flowers and siliques generated a transcriptome of 4.46 G clean data and 31 552 000 of 50-bp pair-end reads, which were assembled into 466 800 contigs. In total, 43.350 unigene sequences were obtained, with a median length of 598bp. This suggests that the transcriptome data represented adequate sequencing depth and genome coverage to meet the requirement of identifying candidate gene sequences expressed in the tissue samples in the current study.

### Sequence and phylogeny analysis

By using all of the annotated family members of *IPT*, *CKX*, *SUT*, *CWINV* and *AAP* in *Arabidopsis* and *Brassica* species available in the GenBank database including a large number of those uploaded after September 2014 (*Brassica rapa* Annotation Release 100) as query sequences to BLAST-search our *B. napus* transcriptome data, we identified most of the putative *IPT*, *CKX, SUT*, *CWINV* and *AAP* orthologous sequences in forage brassica. Results of sequence verification via BLAST-searching the GenBank database showed that most of the identified sequences were confirmed to be the target gene sequences. Multiple sequence alignments and phylogenetic analyses showed that two or three sequences with very high similarity were identified for the majority of the members of the multigene families. Up to four sequences were identified for some family members (Supplementary Table S4). In the case where three or four sequences were identified in the *B. napus* genome, one of the sequences shared high similarity with the orthologue in the C genome of *B. oleraceae*, while each of the others was more similar to an orthologue in the A genome of *B. rapa*.

**Fig. 2.**
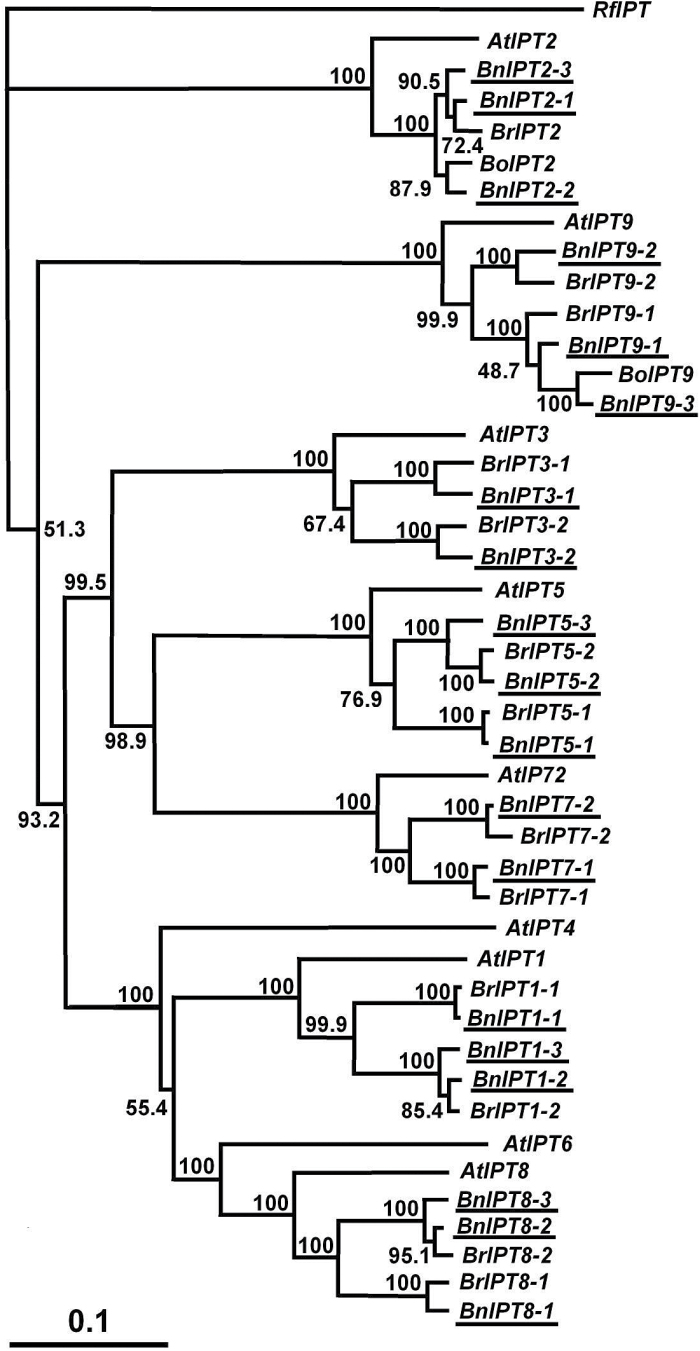
Neighbor-Joining phylogenetic tree for *IPT* gene sequences in *Brassica napus* and closely related species. *At*, *Arabidopsis thaliana*; *Bn, B. napus*; *Bo*, *B. oleraceae*; *Br*, *B. rapa*. The tree was rooted using *Rhodococcus fascians IPT* gene sequence. Node values are percentages of bootstraps generated with 1000 bootstrap replicates. Sequences identified in this study are underlined.

Nineteen sequences for seven of the nine *IPT* gene family members in *B. napus* were identified, with two distinct sequences for *BnIPT5* and *BnIPT7*, and three each for *IPT1, 2, 3, 8* and *9.* Twelve of the sequences were highly similar to their counterparts in the A genome, two to the C genome, and the remaining five may align with the C genome but these sequences are not yet available in GenBank. However, sequences for *BnIPT4* and *BnIPT6* were not found. In addition to the two tRNA-associated *IPT* clades, orthologues of *IPT3*, *5* and *7* formed a distinct clade while those of *IPT1* and *8* formed another clade (Fig. 2).

**Fig. 3.**
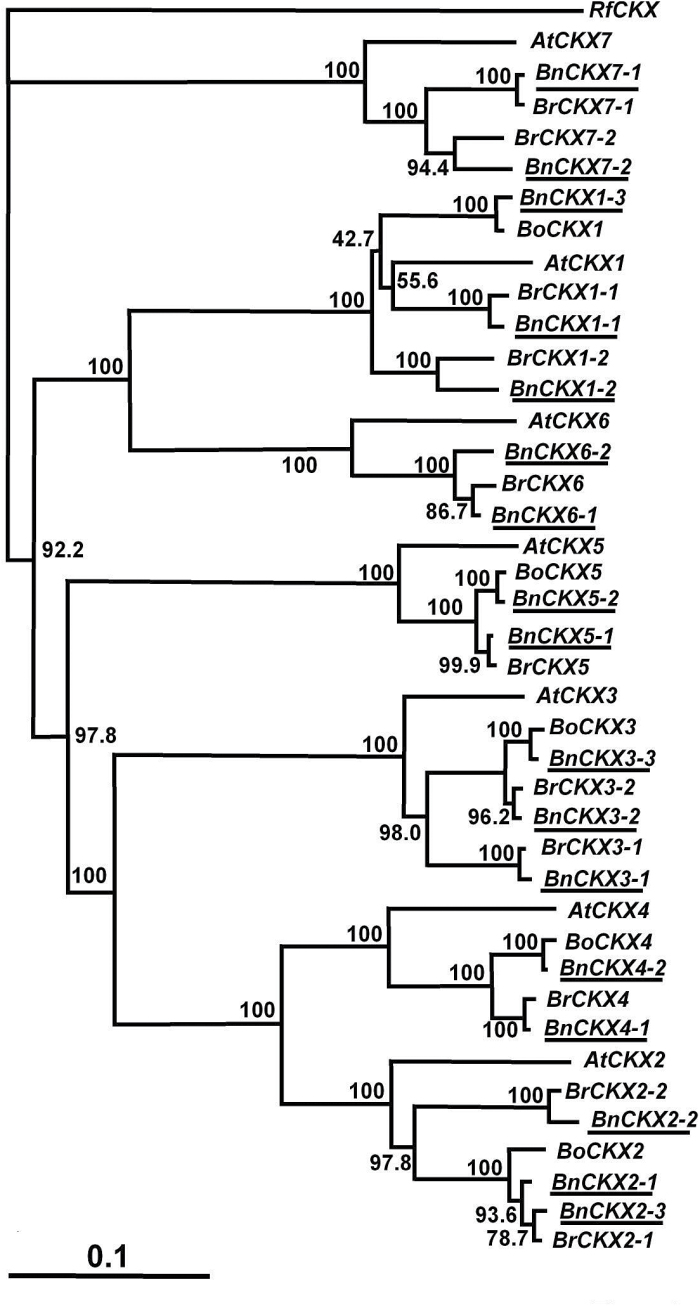
Neighbor-Joining phylogenetic tree for *CKX* family members in *B. napus* and closely related species. The tree was rooted using *Rhodococcus fascians CKX* gene sequence. See Fig. 2 for other information.

Sequences for all the seven *CKX* gene family members in *B. napus* were identified. Two distinct sequences for *BnCKX4, 5, 6* and *7*, and three for *BnCKX1, 2* and *3* were identified (Supplementary Table S4) and allocated to distinct sub-clades with their orthologues from *Arabidopsis, B. rapa* and *B. oleraceae* (Fig. 3).

**Fig. 4.**
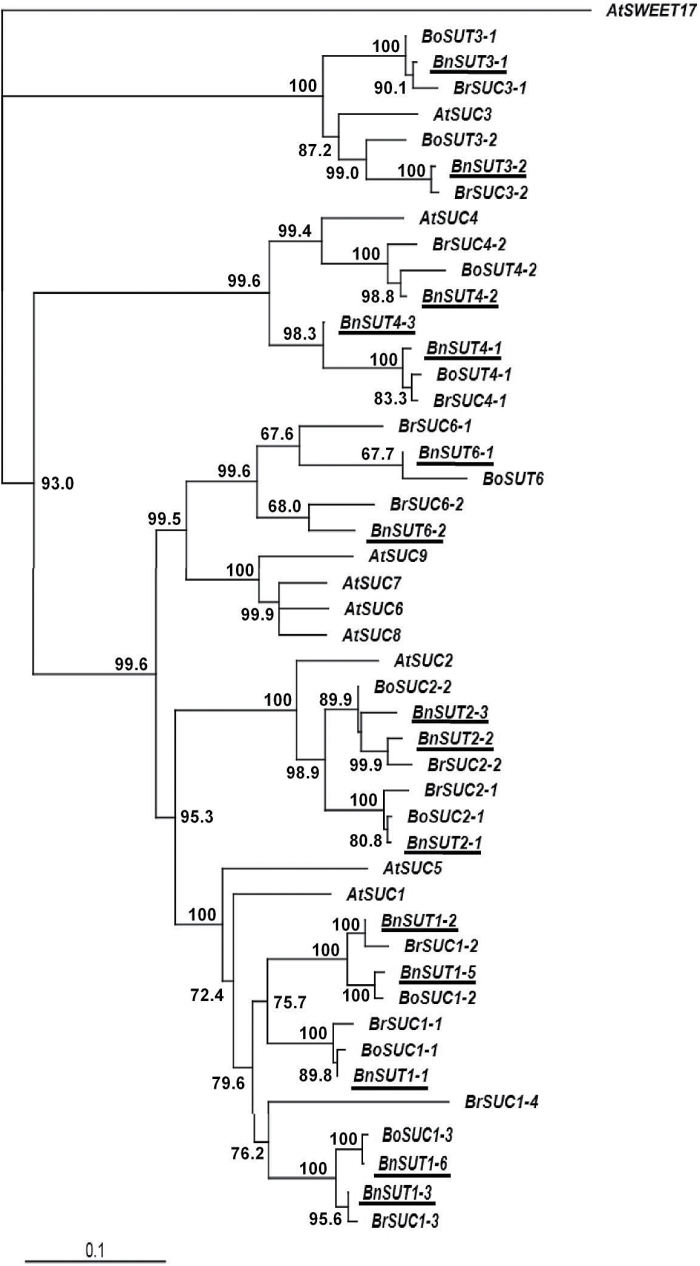
Neighbor-Joining phylogenetic tree for sucrose transporter (*SUT*) gene sequences in *Brassica napus* and closely related species. The tree was rooted using maize *SUT1* gene sequence. See Fig. 2 for other information.

Sequences of *SUT* gene family members were identified from each of the three clades to which *Arabidopsis* and *B. rapa SUTs* are allocated. Duplicate and triplicate sequences were identified (Supplementary Table S4), which grouped together with their orthologues from *B. rapa* or *B. oleraceae* (Fig. 4). No sequences of the *Arabidopsis SUC7*, *8* and *9* orthologues were identified in our transcriptome data, or for *B. rapa* in the current publically available databases.

**Fig. 5.**
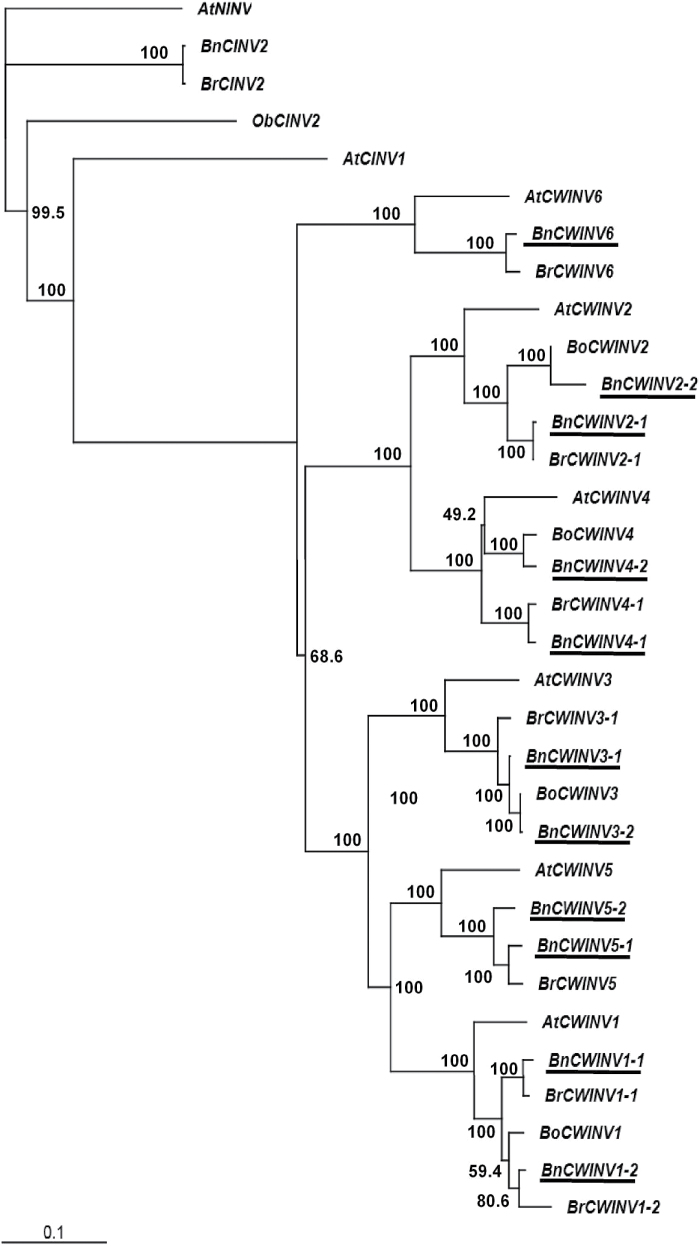
Neighbor-Joining phylogenetic tree for cell wall invertase (*CWINV*) gene family members in *B. napus* and closely related species. The tree was rooted using an *Arabidopsis* alkaline/neutral invertase *AtNINV* gene sequence. See Fig. 2 for other information.

**Fig. 6.**
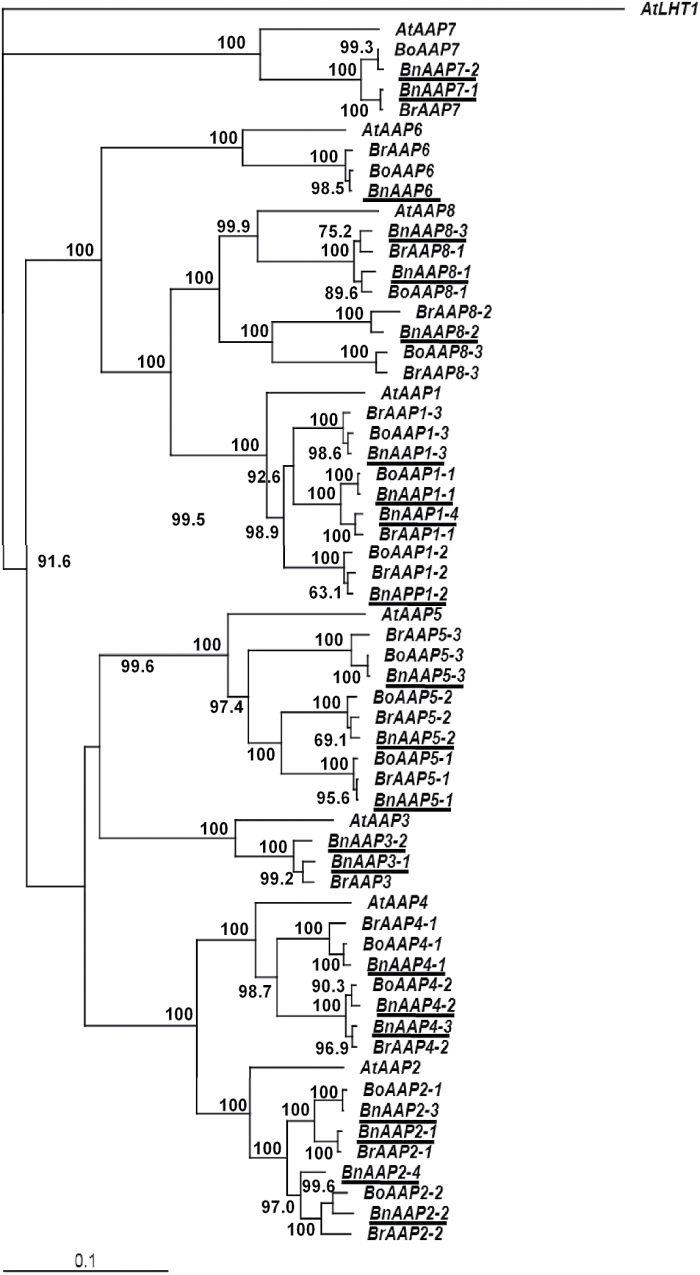
Neighbor-Joining phylogenetic tree for amino acid permease (*AAP*) gene family members in *B. napus* and closely related species. The tree was rooted using a closely related lysine histidine transporter 1(*AtLHT1*) gene sequence. See Fig. 2 for other information.

Six *BnCWINV* members were identified (Fig 5, Supplementary Table S4), with one of the paired sequences sharing high similarity with its orthologue in *B. rapa* and the other with *B. oleraceae* in the case of *CWINV2, 3,* and *4* (Fig. 5). Multiple sequences were identified for most of the AAP family members: four for *BnAAP1* and *2*, three for *BnAAP4, 5* and *8* but only one for *BnAAP6* (Supplementary Table S4). Each sequence allocated well to sequences from either *B. rapa* or *B. oleraceae* (Fig. 6).

### Expression in leaves

**Fig. 7.**
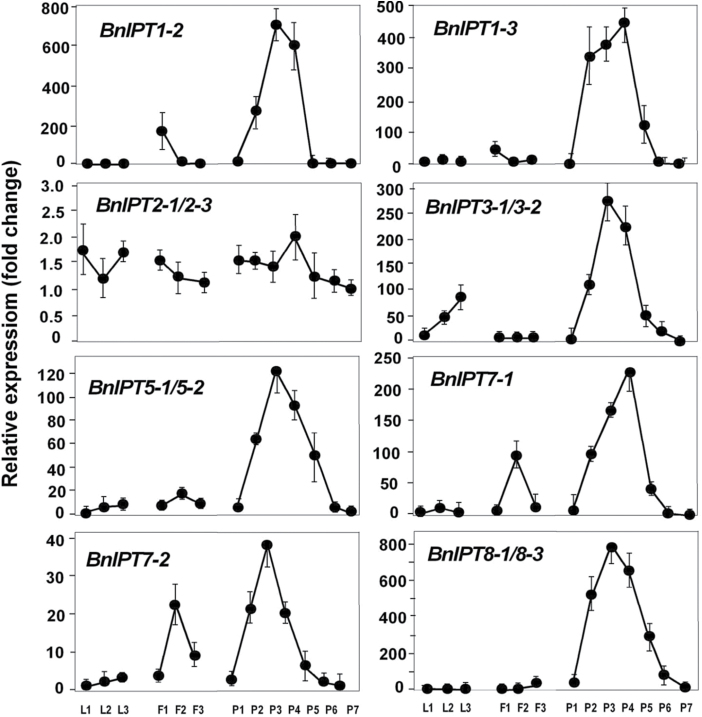
Expression profiles of selected isopentenyl transferase (*IPT*) gene family members in *Brassica napus.* Y-axis values are relative expression (fold-changes) averaged from 3–4 technical replicates calculated relative to the lowest expressed sample of the same sample set, and were corrected using the geometric means of reference genes *α-EF1* and *GAPDH*. Error bars represent standard deviation. L1, young expanding leaves; L2, expanded leaves; L3, senescing leaves; F1, flower buds 3–4mm; F2, flower buds 6–8mm; F3, open flowers; P1–P7, siliques at different developmental stages, from 15–20mm to full size of 70–80mm.

**Fig. 8.**
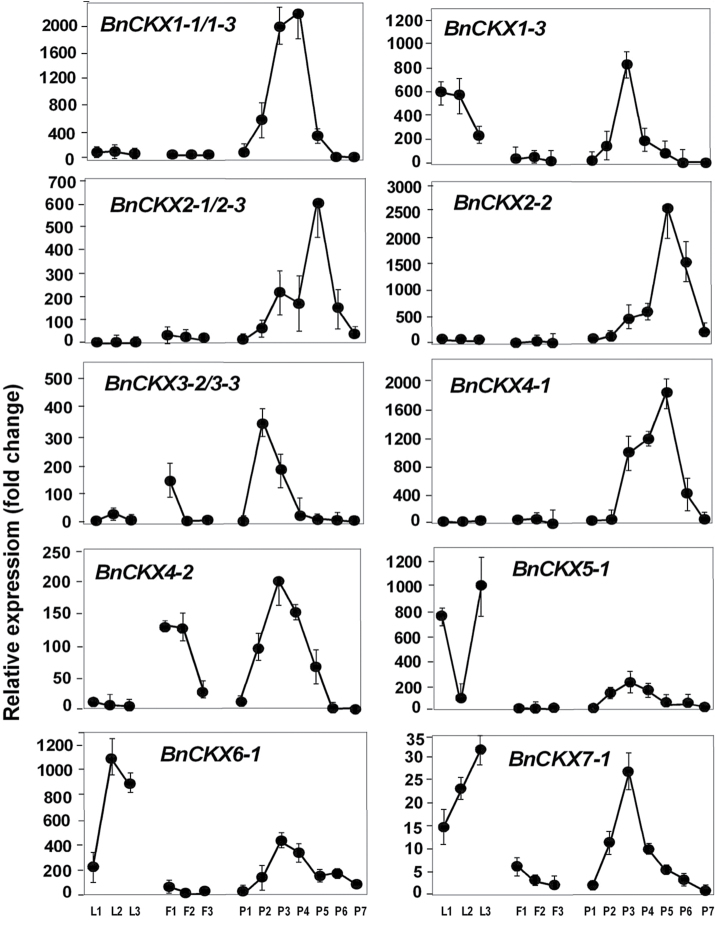
Expression profiles of selected cytokinin oxidase/dehydrogenase (*CKX*) gene family members in *Brassica napus.* See Fig. 7 for legend.

Analysis focused on leaves as expanding sink, mature source and senescing leaves. *BnIPT* expression in leaves was low, particularly relative to developing siliques. Interestingly, *BnIPT3* showed expression in senescing leaves (Fig. 7). Strong differences were seen in expression between *BnCKX* gene family members in the leaves (Fig. 8). Whereas *BnCKX1-3* showed a decreasing expression as leaves aged, *BnCKX5-1* expression was high in sink leaves, reduced in source leaves, and high again in senescing leaves. In contrast, *BnCKX6-1* showed strong expression in both mature and senescing leaves. *BnCKX2-1, 2-2*, and *4-1* showed no expression in leaves at any of the stages examined, and expression of *4-2* and *7-1* was minimal (Fig. 8).

**Fig. 9.**
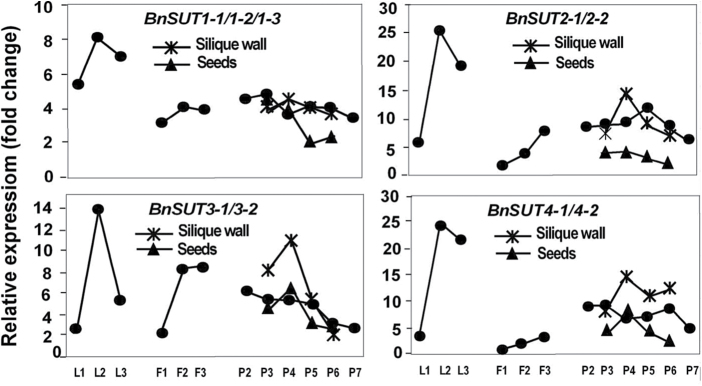
Expression profiles of selected sucrose transporter (*SUT*) gene family members in *Brassica napus.* See Fig. 7 for legend.

**Fig. 10.**
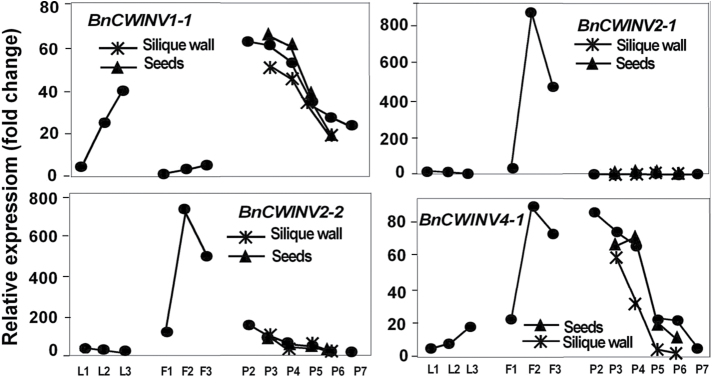
Expression profiles of selected cell wall invertase (*CWINV*) gene family members in *Brassica napus.* See Fig. 7 for legend.

**Fig. 11.**
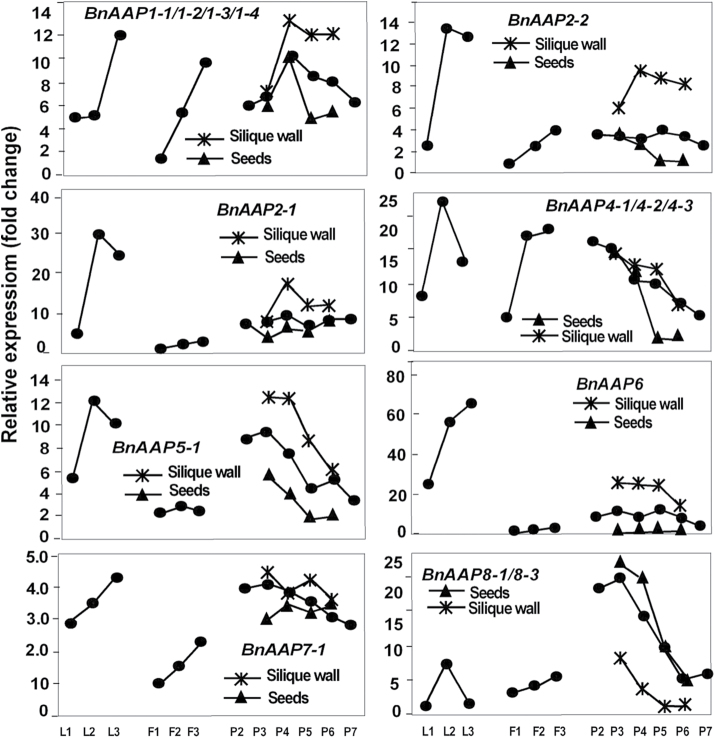
Expression profiles of selected amino acid permease (*AAP*) gene family members in *Brassica napus.* See Fig. 7 for legend.

All four *BnSUT* gene family members showed the same pattern of expression in leaves: relatively low in the young expanding leaves, at a peak in mature leaves, and decreased expression in the senescing leaves (Fig. 9). Differential expression among the cell wall invertase gene family members was evident with increasing expression during leaf development being shown by *BnCWINV1-1*, and to a lesser extent by *4-1*. *BnCWINV2-1* and *2-2* showed little expression in leaves (Fig. 10). Amino acid permease expression was relatively low in expanding leaves but was greater in most cases in the mature source leaves (Fig. 11). The gene family members showed variable expression in senescing leaves with the expression of *BnAAP1, 6* and *7-1* continuing to increase and that of *2-1, 4, 5-1,* and *8* decreasing. Greatest expression overall was shown by *BnAAP6*.

### Expression in flowers

Variation between the *BnIPT* gene family members was evident with little or no expression in flower buds or flowers detected for *BnIPT3, 5* or *8*. *BnIPT1-2* and *1–3* showed expression in the smallest flower buds whereas *BnIPT7-1* and *7-2* showed peaks of expression in the medium-sized flower buds (Fig. 7). Expression of *BnCKX* gene family members varied, with little or no expression detectable for *CKX1-1, 1–3*, *2-1/* 2–3, *2-2*, *5-1*, *6-1* or *7-1*. Both *3-2/3-3* and *4-2* showed elevated expression in small buds, and this was maintained by *BnCKX4-2* as buds expanded. Overall there was minimal expression of *BnCKX* in open flowers (Fig. 8).

As flowers developed there was increasing expression of the *BnSUT* gene family members (Fig. 9). Similarly, the expression of most *BnAAP* gene family members also increased (Fig. 11). Relative to their expression in other plant parts, both *BnINV2-1* and *2-2* were very strongly expressed as flowers developed with peak expression in the larger buds. *BnINV4-1* showed a similar pattern, whereas *1-1* was only weakly expressed (Fig. 10).

### Expression in siliques, silique walls and seeds

*BnIPT* expression rose sharply in P2 siliques, reaching a peak either in P3 or P4 siliques (Fig. 7). *BnIPT1-2* and *1–3*, and *BnIPT8* were the most abundantly expressed gene family members. *BnIPT2* also showed a small change in expression. Most *BnIPT* gene expression had decreased substantially in P5 siliques. Expression of most *BnCKX* family members showed significant changes during silique development: *CKX3-2/3-3* peaked at P2; *CKX1-3, 4-2, 5-1, 6-1* and *7-1* peaked at P3; *BnCKX1-1/1–3* peaked around P3/P4. *BnCKX2-2, CKX2-1/2–3,* and *CKX4-1* peaked at P5. *BnCKX2-2* showed the strongest expression and *BnCKX7-1* showed the weakest (Fig. 8).

**Fig. 12.**
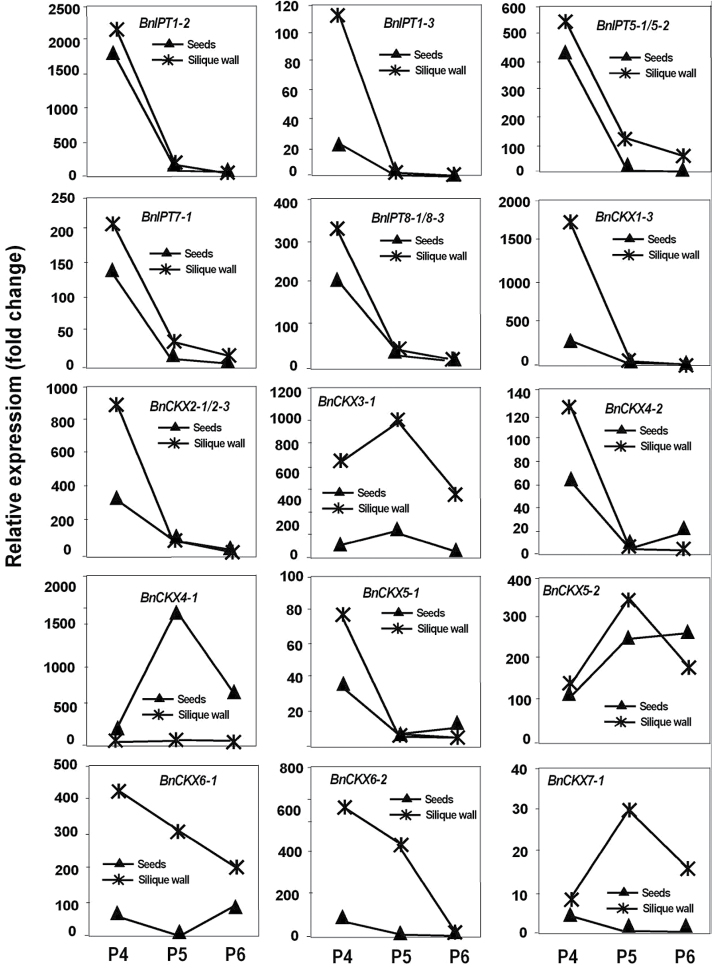
Expression profiles of selected *IPT* and *CKX* gene family members in seeds and silique walls of *Brassica napus.* Y-axis values are fold-changes averaged from 3–4 technical replicates. P4, silique length 50–55mm; P5, silique length 60–65mm; P6, silique length 70–75mm.

It was difficult to cleanly separate seed at the early stages of silique development but, even with some degree of cross contamination allowed for, it is clear that *BnIPT* expression was decreasing rapidly in both siliques and seeds between P4 and P5 (Fig. 12). Much of the *BnCKX* expression was located in the silique walls with the exception of *BnCKX4-1* where peak expression occurred at P5 and was located in the seed.

Transcript expression patterns of the four *BnSUT* family members were not strongly differentiated in the siliques and seeds; expression generally decreased after P4 (Fig. 9). Compared to flowers, the transcripts of *BnCWINV* were fewer, with both *BnCWINV1-1* and *4-1* reducing sharply during development (Fig. 10). Strong differential expression patterns were shown by the *BnAAP* family members. *BnAAP4* and *BnAAP8* were the most strongly expressed in the developing seeds and transcript levels decreased sharply as siliques expanded and seeds developed (Fig. 11).

### Genomic contribution

The contribution of the *B. napus* genes derived from the A genome of *B. rapa* and the C genome of *B. oleraceae* differed quantitatively and in some cases were also differentially expressed on an organ-specific basis. However, no fixed pattern of this genome-specific expression was observed. For example, there was a marked difference in tissue-specific expression of *BnCKX4-1* and *4-2*, with *4-1* (A genome) very strongly expressed in developing seeds, and *4-2* (C genome) strongly expressed in flower buds. *BnCKX5-2* (C genome) was more strongly expressed in silique walls and seeds compared to *5-1* (A genome), as well as developmentally later. Both the *BnCWINV* gene family members expressing in siliques and seeds (*BnCWINV1-1* and *BnCWINV4-1*) derived from the A genome. In contrast, the two homoeologues of *BnCWINV2*, which were highly expressed in developing flowers but which had low expression in leaves, siliques and seeds, derived from different genomes: *BnCWINV2-1* derived from the A genome whereas the *BnCWINV-2* derived from the C genome.

## Discussion

### Phylogenetic and genomic analyses reveal both amplification and loss of gene family members

Whole-genome duplication followed by functional divergence in the event of polyploidization is thought to be a prominent evolutionary force in eukaryotes and a major contributor to evolutionary novelties (Dun *et al.*, 2014). The *Brassica* genomes have undergone a whole genome triplication after speciation from *Arabidopsis* (Lysak *et al.*, 2005), leading to significant expansion of gene numbers. In general, for each ancestral gene in *Arabidopsis*, three syntenic copies could be identified in diploid *Brassica* species such as *B. rapa* (Wang *et al.*, 2011; Cheng *et al.*, 2013; Dun *et al.*, 2014). However, due to genome shrinkage and loss of function of some genes, the triplicated *B. rapa* genome contains only 1.5–2 times the number of genes as in *Arabidopsis* (Mun *et al.*, 2009; Wang *et al.*, 2011). For instance, Liu *et al.* (2013) were able to identify only 13 *IPTs* and 12 *CKXs* from the Chinese cabbage *B. rapa* genome draft, instead of 3 × nine *IPTs* and 3 × seven *CKXs,* respectively. This is also the case for the gene families investigated in the current study.

Multiple sequence comparison and phylogenetic analysis of *IPT*, *CKX*, *SUT*, *CWINV* and *AAP* gene families in forage brassica and its genome contributors revealed that the amplification and/or decrease of gene sequences was family member-specific. In the majority of cases, two sequence variants in *B. rapa* could be identified from public databases for each of the *Arabidopsis* gene family members. This is the case for *IPT1-3*, *5*, *7-9*, *CKX1-3* and *7*, *SUT2-4, 6*, *CWINV1*, and *AAP2* and *4*. However, four sequence variants of *SUT1* and three sequence variants of *AAP1*, *5* and *8* were found in *B. rapa*. These stronger sequence amplifications/retentions may be due to the functional importance of these family members. *SUT1* is a dicot specific Type I sucrose transporter which is suggested to be associated with evolution of vascular cambium and phloem transport (Peng *et al.*, 2014). In contrast, sequence variants in some family members may have decreased or even been completely lost during evolution due to loss of function. For instance, no sequences of *IPT4* and *6*, or of *SUT5, 7, 8* and *9* were identified from our transcriptome data, or from the *B. rapa* genome draft by Li *et al.* (2013) and Peng *et al.* (2014).

*B. napus* is a tetraploid containing A and C genomes derived from *B. rapa* and *B. oleraceae*, respectively. While there is limited sequence data available for *B. oleraceae*, we expected to identify two homoeologues for each sequence variant in *B. rapa.* However, this was true only for *CKX4-6*, *CWINV2-5*, and *AAP2*, *3* and *7*, for which we were able to identify two sequence variants in *B. napus* for each *B. rapa* sequence variant. In most cases, fewer than twice the sequence variants were identified. In some extreme scenarios, only one *B. napus* sequence was identified for each *B. rapa* sequence. This was the case for *IPT3*, *7*, *CKX7*, *SUT3*, *6*, *CWINV1*, *6*, and *AAP5*, *6* and *8*. As there is no whole genome sequence available for forage brassica we were not able to determine the actual reason for this sequence decrease. We suggest that this may be due to several factors including the fact that these variants may not have been expressed in our RNA-seq sample, or were not detectable due to low sequencing depth, or, more likely, due to loss of function or complete loss of gene sequences, particularly from *B. oleraceae*.

### Silique and seed development occurred concurrently

Using terminology as defined by Locascio *et al.* (2014), seeds from P1–P4 siliques were at the first phase of seed development—morphogenesis—with the seed undergoing endosperm development, cell divisions, and embryo and cotyledon differentiation. Seeds from P5–P7 siliques were at the second phase of development—maturation—including embryo growth by cell expansion, absorption of the endosperm by the embryo and dry matter accumulation.

However, the seeds were not fully mature as they had not begun to desiccate (Locascio *et al.*, 2014). As microscopic dissection of seeds from P1, 2 and 3 siliques failed to detect a developing embryo, most metabolic activity in these seeds would be associated with the syncytial cell divisions occurring in the endosperm. As heart and torpedo stage embryos were present in seeds from P4 siliques, it is likely, as in *Arabidopsis* and oilseed rape, that cellularization of the endosperm was essentially completed in P4 siliques, and that cell proliferation in seeds from P5 siliques was complete as the cotyledons were expanding and the endosperm was being absorbed (Morley-Smith *et al.*, 2008; Dante *et al.*, 2014).

However, competition for resources between the elongating silique and the seeds inside was likely to be occurring as the siliques were steadily elongating during both phases of seed development. As the siliques were green they potentially contributed to carbohydrate metabolism, as shown for *B. napus* siliques (King *et al.*, 1997), but competition for nitrogen would have been occurring.

### Silique walls and seeds are active sites for cytokinin biosynthesis

While it was established early on that filial tissues of the developing seed of legumes were reliant on *in situ* cytokinin biosynthesis (e.g. Jameson *et al.*, 1987; Singh *et al.*, 1988; Letham 1994), this was less clear for pod walls and seed coats as both were shown to have significant amounts and diversity of cytokinin forms (Davey and van Staden, 1977, 1978, 1979; Summons *et al.*, 1981; Zhang and Letham 1990), and were in receipt of xylem- and/or phloem-supplied cytokinin (Jameson *et al.*, 1987; Singh *et al.*, 1988; Taylor *et al.*, 1990; Zhang and Letham, 1990).

Differential spatio-temporal expression patterns of *IPT* gene family members have been shown in *Arabidopsis* (Miyawaki *et al.*, 2004; Belmonte *et al.*, 2013) and other Brassicaceae: in Rapid Cycling *B. rapa* (RCBr) (O’Keefe *et al.*, 2011) and in Chinese cabbage (*B. rapa* ssp. *perkinensis*) (Liu *et al.*, 2013). Using RT-PCR, Miyawaki *et al.* (2004) showed, between all seven *AtIPT* gene family members, that cytokinin biosynthesis could occur in most organs of the plant. However, using the GUS reporter gene construct, quite distinct tissue specificity was shown although each family member expressed at more than one location. Liu *et al.* (2013) showed that while *BrIPT1, 3, 5* and *7* were most strongly expressed in the roots, *BrIPT8-1* was expressed in immature siliques, and *BrIPT8-2* in stamens. This aligns well with *Arabidopsis*, where *AtIPT8* (but also *AtIPT4*) was shown to be localized to the chalazal region in developing *Arabidopsis* seeds (Miyawaki *et al.*, 2004; Day *et al.*, 2008; Belmonte *et al.*, 2013), but with significantly decreased expression in seeds at the linear and mature green cotyledonary stage (Belmonte *et al.* 2013).

In forage brassica *BnIPT8* was strongly expressed in the developing siliques, and transcript levels were reduced to low levels during seed maturation. However, unlike *Arabidopsis* (Miyawaki *et al.*, 2004; Belmonte *et al.*, 2013), neither homoeologue of *BnIPT4* was shown to express in any of the tissues analysed, whereas *BnIPT1-2/1-3* was as strongly expressed as *BnIPT8-1/8-3* in siliques, and more so in developing seeds. *AtIPT1* was detected in siliques and in the integument and seed coat of immature *Arabidopsis* seeds (Miyawaki *et al.*, 2004), but *BnIPT1* from Chinese cabbage was not detected in immature siliques (Liu *et al.*, 2013). It is noteworthy that neither *IPT4* nor *6* were detected by us, Ando *et al.* (2005) or Liu *et al.* (2013) in Chinese cabbage (*B. rapa*). While *AtIPT6* and *OsIPT6* are regarded as pseudogenes in some accessions of *Arabidopsis* and cultivars of rice respectively (Kakimoto *et al.*, 2001), clearly the loss of *IPT4* from *Brassica* sp. indicates that *Arabidopsis* is not an infallible model for gene expression in the Brassicaceae.

What is clear from forage brassica is that both silique wall and seed are capable of cytokinin biosynthesis, but that this capability has declined in both organs by the end of phase one. In forage brassica, cytokinin biosynthesis is, therefore, associated with the morphogenesis phase of free nuclear divisions, cell division and differentiation. Notable also is that *BnIPT* expression was not detected in the smallest of the siliques sampled, leaving those dependent on a maternal source of cytokinin, as suggested for white lupin where xylem/phloem accounted for most of the cytokinin supply during early pod set (Emery *et al.*, 2000).

While a degree of tissue specificity was shown for the expressed *BnIPT* family members, tRNA-associated *BnIPT2* was expressed at a low level in the leaves, flower buds, flowers and siliques, but with slightly more transcript measured during the stages of rapid silique and seed development. Based on the fact that the tRNA-associated IPTs are constitutively expressed and the functional similarity between *IPT9* and *IPT2*, *BnIPT9* expression in forage brassica was not investigated in this work although two homoeologue gene sequences were identified from our transcriptome data. This is in agreement with Miyawaki *et al.* (2004), who showed that both *AtIPT2* and *9* were expressed constitutively, but more strongly in proliferating tissues (Miyawaki *et al.*, 2004). Liu *et al.* (2013) also showed low, constitutive expression of both *BrIPT2* and *9* in Chinese cabbage.

### CKX limits endogenous cytokinin levels during development

Cytokinin oxidases/dehydrogenases (CKX) catalyse the irreversible degradation of the active cytokinins, isopentenyladenine and zeatin, and their ribosides (Werner *et al.*, 2006). Tissue-specific expression has been reported for both *Arabidopsis* (Werner *et al.*, 2006) and *B. rapa* (Liu *et al.*, 2013), the latter reporting differential expression of *BrCKX* gene family members across a variety of tissues, although the peaks of *BnCKX* activity that were detected in developing siliques and seeds of forage brassica would not have been observed by Liu *et al.* (2013). While *BrCKX2-2* was the most highly expressed in Chinese cabbage but limited predominantly to reproductive tissues, *BnCKX2-1* and *2-2* expression in forage brassica was restricted to siliques and seeds.

Hirose *et al.* (2008) diagrammatically summarized the expression of *AtIPT* gene family members (from Miyawaki *et al.*, 2004) and *AtCKX* gene family members (from Werner *et al.*, 2003) and showed that the expression patterns of *AtIPT* and *AtCKX* gene family members often overlapped. There are now numerous reports suggesting that whenever the expression of the *IPT* genes occurs, or the endogenous cytokinin levels are elevated, expression of *CKX* genes and/or increased CKX activity occurs (O’Keefe *et al.*, 2011 and references therein; Liu *et al.*, 2013). Indeed, there are suggestions that CKX activity acts to limit seed development (Brugière *et al.*, 2003; Li *et al.*, 2013) or has a controlling influence on seed yield (Bartrina *et al.*, 2011). This is a situation reflected in forage brassica, where the expression of many of the *BnIPT* and *BnCKX* gene family members appears to be co-ordinately controlled during silique and seed development. The silique- and seed-specific expression of *BnCKX2*, and that also of *BnCKX4,* make these gene family members our likely targets for mutant selection and functional MAS, along with the fact that AtCKX2 was identified as an integrator of genetic and epigenetic regulation of endosperm growth (Li *et al.*, 2013).

However, in leaves, biosynthesis of cytokinin appears relatively limited. We show, with the exception of *BnIPT3* in mature and senescent leaves, that there is limited *BnIPT* expression in leaves, as also shown for maize by Vyroubalová *et al.* (2009) and in Chinese cabbage by Liu *et al.* (2013). Consequently, a supply of cytokinin from the roots to leaves is implicated (Letham 1994; Hirose *et al.*, 2008) and the strong *BnCKX* expression detected in sink (*BnCKX1-3* and *4-1*), source (*BnCKX1-1* and *6-1*) and senescing forage brassica leaves (*BnCKX5-1* and *6-1*) can be interpreted as a response to the presence of endogenous cytokinin in developing and mature leaves, and the destruction of cytokinin to enable the final remobilization of resources from senescing leaves to the sinks (Werner *et al.*, 2006).

In forage brassica flowers, as in leaves, there was limited expression of *BnIPT*, and what there was in the flower buds was matched by *BnCKX4-2* expression. Similarly, in Chinese cabbage, while there was limited *BnIPT* expression in flowers there was detectable *BnCKX* expression (Liu *et al.*, 2013). This supports the contention that the developing flower and ovule is also dependent on maternally supplied cytokinin, in contrast to the expanding silique and developing seeds, but with CKX providing a moderating influence in all tissues.

### Sucrose transporters are active in multiple tissues

SUTs belong to a small gene family and are essential for the export and efficient movement of sucrose from source leaves to sink organs (Peng *et al.*, 2014). They function to load sucrose into the phloem and to unload sucrose into sink tissues such as seeds and flowers (Reinders *et al.*, 2012). Braun *et al.* (2014) suggested that the function of the plasma membrane-located SUTs is critical in apoplastic loading species. Based on our multiple sequence alignment and phylogenetic analysis, forage brassica BnSUT1, 2 and 3 are most likely plasma membrane-located, and BnSUT4 tonoplast-located (Fig. 4). Li *et al.* (2011) reported that rapeseed *BnSUT1* is associated with a QTL for yield. Spatial and developmental profiling of the rapeseed *BnSUT1* showed abundance of transcript in source leaves and stems, and lower levels in reproductive organs. However, in these latter organs, *BnSUT1* was more strongly expressed in the pistil and when the siliques were rapidly elongating, reducing to lower levels when the dry weight of the siliques reached a maximum (Li *et al.*, 2013). This pattern is reflected not only by forage brassica *BnSUT* expression in the developing siliques and seeds but also in the sharp increase in expression as leaves developed from being sinks to being source leaves, and subsequently senescing. The requirement of developing flowers for sucrose is reflected also by an increase in *BnSUT* expression.

Interestingly, the heat map compiled by Peng *et al.* (2014) shows high activity for only three of the seven Type I *AtSUTs,* with *AtSUC2* being expressed in both vegetative and reproductive tissues, *AtSUC1* similarly but only in tissues in which *AtSUC2* was not expressed, and *AtSUC9* expression restricted to pollen tubes (in which neither *AtSUC2* nor *AtSUC1* were expressed). The single Type II and III *AtSUTs* showed substantially lesser expression. Similarly, in forage brassica the number of expressed Type I *SUTs* is also limited as only *BnSUT1, 2* and *6* sequences were identified from the transcriptome data. However, the Type I, Type II and III *BnSUTs* showed relatively similar expression patterns to each other, both in terms of organ expression and developmentally.

### Cell wall invertase gene family members show strong tissue specificity

Ruan *et al.* (2010) suggested that invertase has a wide range of regulatory functions in plant growth and development in addition to its major role in primary carbon metabolism, and that CWINV is essential for flower, seed and fruit production and may even be rate-limiting to seed development through its effects on cell division in filial tissue. Wang and Ruan (2012) showed *AtCWIN4* to express more strongly than *AtCWIN2*, and to express both in the syncytial endosperm near the chalazal region as well as in the embryo from globular to torpedo stage. The transition from cell division and expansion to storage activities in seeds is usually associated with a decrease in invertase expression and activity (Wang and Ruan, 2013). The expression profile of forage brassica *BnCWINV1* and *4* in seeds is in accord with this. However, the strongest expression of forage brassica *BnCWINV* was of both homoeologues of *BnCWINV2* in developing and open flowers, suggesting that BnCWINV is supplying hexoses, possibly to the developing anthers and ovaries (Ruan *et al.*, 2010) and expanding petals (Bihmidine *et al.*, 2013).

### Amino acid permease gene family members are spatio-temporally differentiated

Activity of *AAP* gene family members has been shown variously in roots, xylem parenchyma, transport phloem and sink tissues including cotyledon and endosperm (Tegeder, 2014). Tegeder (2012) assigned the eight *Arabidopsis* gene family members to three clusters. *AtAAP2, 3, 4* and *5* were all assigned to Cluster 3A, and all are considered to be transporters generally associated with loading into the phloem: AtAAP3 function was restricted to the roots; AtAAP5 was associated with loading into the companion cells of both roots and leaves; AtAAP4 with leaf phloem loading and AtAAP2 with phloem loading along the transport pathway (reviewed by Tegeder, 2012). Expression of *BnAAP3* was not detected in forage brassica with the implication that it may also be confined to root expression in this species. However, transcripts for *BnAAP2-1, 2-2*, *4-1/4-2/4-3* and *5-1* were detected not only in leaves, but also in developing flower buds, open flowers, siliques and developing seeds, implicating these transporters not just in phloem loading but also in importation into sink tissues.

Gene family members *AtAAP1, 6* and *8* are in cluster 4B (Tegeder, 2012). While *AtAAP6* has been associated with exchange of nitrogen between xylem and phloem, *AtAAP1* and *8* have been associated with seed loading (reviewed by Tegeder, 2012). In forage brassica, transcript levels of *BnAAP8* were greatest at the earliest stages analysed and reduced sharply as seeds developed implying a similar role in early seed development to AtAAP8.

Tilsner *et al.* (2005) isolated and characterized three amino acid permeases, *BnAAP1, BnAAP2,* and *BnAAP6,* in rape seed. With work focused on the mobilization of nitrogen reserves from senescing leaves, Tilsner *et al.* (2005) showed that all three transporters were expressed in leaves and the expression was still detectable during leaf senescence, with *BnAAP1* and *BnAAP2* mRNA levels increasing from mature to old leaves. In leaves of forage brassica, most of which are senescent at the later stages of silique maturity, expression of *BnAAP1, 2* and *6* increased or stabilized between mature and senescing leaves. Expression of *AAPs* in mature and senescing leaves is not unexpected as these are the main source of nitrogen for the developing siliques and seeds. However, the decrease in expression of the *BnAAPs* in seeds before the main phase of storage protein accumulation indicates transporters other than AAPs must be active.

Tegeder (2012) does note that the expression of *AtAAPs* generally is not that phloem- or seed-specific, and the data for forage brassica are in agreement with this, indicating a high degree of functional redundancy throughout much of the plant.

## Conclusions

We have shown how a single transcriptome of mixed tissues can provide the information to detect expressed genes for subsequent profiling work. While the amplification of genes within clades provides a phylogenetic challenge, careful primer design allows for the detection of expression of individual gene family members and their homoeologues.

The co-ordinated import of sucrose and amino acids is required for successful silique and seed development and requires the integrated expression of different genes (Zhang *et al.*, 2010). However, Morandini (2013) suggests that: ‘if one desires to increase [metabolic] flux, it seems more sensible to try to increase sink strength rather than tampering with signals or signal transduction events’. To increase sink strength requires an increase in seed number and/or seed size. The *CKX* gene family are an appropriate target as potentially *BnCKX* restricts *BnIPT* activity during and to the first phase of seed development.

A direct consequence of decreased CKX activity in the silique and seed is anticipated to be increased cytokinin levels. An indirect consequence should be increased CWINV activity, providing the sugar signals for enhanced cell cycle activity. The strong positive correlation between expression of *BnIPT* and two *BnINV* gene family members identifies these as good markers for such a response. Clear differential gene expression within the *BnAAP* gene family members as well as identification of the four expressed *BnSUC* family members allows for these to be used as markers for quality changes in the plant following the selection and breeding of plants with mutant *CKX2* and/or *4* genes, identified from TILLING and EcoTILLING populations.

## Supplementary Data

Supplementary data are available at *JXB* online.

Supplementary Table S1. GenBank accession numbers of the gene sequences isolated in this study.

Supplementary Table S2. Sequences of qPCR primers used in this work (F, forward; R, reverse).

Supplementary Table S3. Gene expression profiles during development (Biological replicate 2).

Supplementary Table S4. Gene family member(s) which could be amplified by the primer pairs used in this work.

Supplementary Data

## Abbreviations:

MAS: marker assisted selection
QTL: quantitative trait loci
TILLING: targeting induced local lesions in genomes
RT-qPCR: quantitative reverse transcription polymerase chain reaction.
