# Supplementary material for: Expression patterns of Brassica napus genes implicate IPT, CKX, sucrose transporter, cell wall invertase, and amino acid permease gene family members in leaf, flower, silique, and seed development
Source: J Exp Bot. 2015 Apr 4;66(16):5067–82. doi: 10.1093/jxb/erv133 (PMC4513924; doi:10.1093/jxb/erv133)
Supplement: Supplementary Data [file supp_66_16_5067__index.html]

Expression patterns of Brassica napus genes implicate IPT, CKX, sucrose transporter, cell wall invertase and amino acid permease gene family members in leaf, flower, silique and seed development — Expression patterns of Brassica napus genes implicate IPT, CKX, sucrose transporter, cell wall invertase and amino acid permease gene family members in leaf, flower, silique and seed development — Expression patterns of Brassica napus genes implicate IPT, CKX, sucrose transporter, cell wall invertase, and amino acid permease gene family members in leaf, flower, silique, and seed development — Supplementary Data 

# Expression patterns of *Brassica napus* genes implicate *IPT, CKX*, sucrose transporter, cell wall invertase, and amino acid permease gene family members in leaf, flower, silique, and seed development

## Supplementary Data

Data files

**Files in this Data Supplement:**

- Supplementary Data - Supplementary Data
