# Supplementary material for: Expression patterns of Brassica napus genes implicate IPT, CKX, sucrose transporter, cell wall invertase, and amino acid permease gene family members in leaf, flower, silique, and seed development
Source: J Exp Bot. 2015 Apr 4;66(16):5067–82. doi: 10.1093/jxb/erv133 (PMC4513924; doi:10.1093/jxb/erv133)
Supplement: Supplementary Data [file supp_erv133_jexbot144857_file001.pdf]

Title: Expression patterns of Brassica napus genes implicate IPT, CKX, sucrose transporter, cell wall invertase and amino acid permease gene family members in leaf, flower, silique and seed development

Authors: Jiancheng Song, Lijun Jiang, and Paula E Jameson

Table S1 GenBank Accession Numbers of the gene sequences isolated in this study

Genes	Accession Numbers.	Genes	Accession Numbers
BnIPT1-1		BnIPT1-2	
BnIPT1-3		BnIPT2-1	
BnIPT2-2		BnIPT2-3	
BnIPT3-1		BnIPT3-2	
BnIPT5-1		BnIPT5-2	
BnIPT5-3		BnIPT7-1	
BnIPT7-2		BnIPT8-1	
BnIPT8-2		BnIPT8-3	
BnIPT9-1		BnIPT9-2	
BnIPT9-3		BnCKX1-1	
BnCKX1-2		BnCKX1-3	
BnCKX2-1		BnCKX2-2	
BnCKX2-3		BnCKX3-1	
BnCKX3-2		BnCKX3-3	
BnCKX4-1		BnCKX4-2	
BnCKX5-1		BnCKX5-2	
BnCKX6-1		BnCKX6-2	
BnCKX7-1		BnCKX7-2	
BnCKX7-3		BnSUT1-1	
BnSUT1-2		BnSUT1-3	
BnSUT1-5		BnSUT1-6	
BnSUT2-1		BnSUT2-2	
BnSUT2-3		BnSUT3-1	
BnSUT3-2		BnSUT4-1	
BnSUT4-2		BnSUT4-3	
BnSUT6-1		BnSUT6-2	
BnCWINV1-1		BnCWINV1-2	
BnCWINV2-1		BnCWINV2-2	
BnCWINV3-1		BnCWINV3-2	
BnCWINV4-1		BnCWINV4-2	
BnCWINV5-1		BnCWINV5-2	
BnCWINV6		BnAAP1-1	

BnAAP1-2	BnAAP1-3
BnAAP1-4	BnAAP2-1
BnAAP2-2	BnAAP2-3
BnAAP2-4	BnAAP3-1
BnAAP3-2	BnAAP4-1
BnAAP4-2	BnAAP4-3
BnAAP5-1	BnAAP5-2
BnAAP5-3	BnAAP6
BnAAP7-1	BnAAP7-2
BnAAP8-1	BnAAP8-2
BnAAP8-3	

Table S2 Sequences of qPCR primers used in this work (F, forward; R, reverse)

Name	Sequence (5'-3')
BnIPT1aF	CGTCCTTCTCCTCACATTCCCTT
BnIPT1aR	GAGTGGCGAGATCGACTGAAAGA
BnIPT1bF	GGGCATGTTCGAGGAGCTGT
BnIPT1bR	CTTCCATACTTGCCTCCTCGCTA
BnIPT2F	ACGTATCTCCCAGACACAAATAGCTC
BnIPT2R	TGTTGCATCAACGTGATGGATATTC
BnIPT3F	GCTTCCAATCATCGTTGGAGGTT
BnIPT3R	TCTCTAGCTTCTTCGACCATTCCAT
BnIPT5F	GACGAGGTTCGCCGCATCTT
BnIPT5R	GGTGCATGTTCCACTTCCACTG
BnIPT7aF	GCTCCACGAGTACTTACGGTACGAATC
BnIPT7aR	GACGATTCTCTTTGTTGGTCTCGCTA
BnIPT7bF	ACCGAAGCAGGTACAACCAATCT
BnIPT7bR	YCCGAGCAAGTGGTGAGGCA
BnIPT8F	GAATGTTCGAGGAGCTCGCAAGT
BnIPT8R	TTCCTCGTACGCAACCTTTCTTG
BnCKX1aF	CCACAGACAAAACAACAAGACTTTCCTC
BnCKX1aR	GCCAAAGGTGGGAACTGGTATCT
BnCKX1bF	CCTTTCTCCCCTCTGCAGTGCA
BnCKX1bR	GCCTTTTGGAAGATTCTGTGACCA
BnCKX2aF	AAGCTGTATCAAATCGTCAGAGATTATTC
BnCKX2aR	GTAGGTGAGGAGACGGGATATCTCAA
BnCKX2bF	CGTCGGAGGAACGCTGTCGA
BnCKX2bR	TGGCTCTCGTTATTATTCCAAATTGAC
BnCKX3aF	ATTCGGAATTATAACAAGAGCCAGGA
BnCKX3aR	TTTATCATTGAGACGATCCTCAAGTGA
BnCKX3bF	AGCTAAAACTGAAATCCAAAGGCAAG
BnCKX3bR	GTCGACATTCGATCATTCCACTTGT
BnCKX4aF	GAGAATCTTAACGACAGGATAATCAACT
BnCKX4aR	CGTTGTATGTTTAACTAAAGATGTCTTGTC
BnCKX4bF	CTGGAATCGGCGGTGAAGTCT
BnCKX4bR	CTGTGAAGTCACTGTACAGCATTCGA
BnCKX5aF	ACTTCACCTTCAAGCCTTCCGACT
BnCKX5aR	CTCATCTCCACCACCACACCGTT
BnCKX5bF	CGATCCACGGCATATACTCGCTAC
BnCKX5bR	CCAACCTAATCTCTCTCTGATGCTTCT

Name	Sequence (5’-3’)
BnCKX6aF	TGGCGATCCTTACATCGGCTGT
BnCKX6aR	TGAGGAGTGTGGTGAGACTACTGGTGA
BnCKX6bF	CACCTCCTTACTACTACTCAGCTGCATAG
BnCKX6bR	ATCACCGAGGCGATGTCGCTTA
BnCKX7aF	GTTAGACGTTGTGACGGGAAATGGA
BnCKX7aR	GTGGTCTGGGTGGAGTGGAACTGTA
BnCKX7bF	GGAGAATCATTTCGAGGTTGGACAT
BnCKX7bR	GTCCCCATTTCCCGTCACCACAT
BnSUT1aF	CCTCACAAATGGTCCTCTCTCATCTG
BnSUT1aR	GTTGTTRGCNACGTCGAGGATC
BnSUT1bF	TCCCGTCTCCGGCATGATTGT
BnSUT1bR	CGACTCGCGTTCTTTTAGCGTCT
BnSUT2aF	CGAACAACACTCTCCAAGGACCATG
BnSUT2aR	TCCATGGCTTCTCCKTCACGTAAC
BnSUT2bF	TCGGGTTYTGGATTCTCGACGTG
BnSUT2bR	TGACCAAGAGGAGTAGCGTTATGGAG
BnSUT3aF	GATCAGCGGAATACTGCAAATGCTG
BnSUT3aR	TGTGACAAGAGTGCATATAGTGAGAAAGAC
BnSUT3bF	ACCTGCTCGTGCTCTTCTAGCTG
BnSUT3bR	AGAAGAAAAGCTGCTTTRAGATTTCCACATG
BnSUT4aF	GTCAGCATTCTACATAGACGTAGTCTTCATC
BnSUT4aR	ACGGAAACCAACCAATCCACGTC
BnSUT4bF	TCTTCATCGCAATAACCACCATCCTAAG
BnSUT4bR	GTAGATCTCTCKACCCATCCAATCAGTATC
BnCWINV1aF	GCTCTGATGCCAAGCCTTCAACTC
BnCWINV1aR	CAAACAAATGAGCGTTGTCACCTACAG
BnCWINV1bF	GCCAAGCCTTCAACTCTCAAAGGAG
BnCWINV1bR	CAGGCTGTGACCCATTGTTGAAGAC
BnCWINV2aF	GCTCTGATGCCAAGCCTTCAACTC
BnCWINV2aR	CAGGCTGTGACCCATTGTTGAAGAC
BnCWINV2bF	CTTCAAAGACACTTCTACAGATAAGCCTAAG
BnCWINV2bR	TTCACTGCTTTCAATGGATACACTCTTG
BnCWINV3aF	CAAGAGCAGAGATTTCTTCAACTGGAC
BnCWINV3aR	GTAAACATCCTTCTCACGGTCGTAAC
BnCWINV3bF	AGCCTCTTCACCACGAAGACTTAAC

Name	Sequence (5’-3’)
BnCWINV3bR	GAGCCGAACCATCTTGCACAAAC
BnCWINV4aF	AGACTATACCATGAATGGTTCAGCCTTC
BnCWINV4aR	TTTGGAGTGAACCGGGTGCTTAC
BnCWINV4bF	GATTAAACCAGATGATAATCCGGTTGCA
BnCWINV4bR	GTATTTGCCAAGCGTGTAATACTCGTAC
BnAAP1aF	TACGGCGAGTGCGCATATTATCAC
BnAAP1aR	CACGGTAGCAATCAGCGAGCATC
BnAAP1bF	CTATGATATTCCCTTTCTTCAACGCCATC
BnAAP1bR	TGTATGTCTTGACACTACTTATCAGTCCTG
BnAAP2aF	GCTGGACCTGCGGTGATGCT
BnAAP2aR	ACTGAATCAGCCCACAAATCTTGAACTTG
BnAAP2bF	CGGCAAGAGAAACTACACTTACATGGAC
BnAAP2bR	ACGGGTCTTTTCCTCCGCTCTTG
BnAAP3aF	CACTTCTCAGTGACTGCTACAGAAC
BnAAP3aR	CCTCCGCTCTTGTGGAAACAG
BnAAP3bF	CACTTCTCAGTGACTGCTACAGAAC
BnAAP3bR	CTTGTGGAAACAGTTCGATCTCTTG
BnAAP4aF	GCTGGACCTGCGGTGATGCT
BnAAP4aR	ACTGAATCAGCCCACAAATCTTGAACTTG
BnAAP4bF	CGGCAAGAGAAACTACACTTACATGGAC
BnAAP4bR	ACGGGTCTTTTCCTCCGCTCTTG
BnAAP5aF	CAAAGAAATCAAAGGCACTCTCACTG
BnAAP5aR	ACTTATGAGAGTCGCTTTCCTCATC
BnAAP5bF	GAGTCACCATTGGGACAGTGAC
BnAAP5bR	TGCATACCCTACGCAGCCACAG
BnAAP6F	CATAATGTGAAATGTTCCACTTCAAACACTC
BnAAP6R	ACTCCGACCGTAACTCCTGTCAG
BnAAP7aF	AACGGTGACACTGAGTCCAATGAG
BnAAP7aR	CTTCTTCTGCAATATACACATCTCCACAG
BnAAP7bF	GACATGCTTTGGCTCTCTGTTGTC
BnAAP7bR	CCCAAGAGCTTGGAACACTAACCATAC
BnAAP8aF	CAAGTCTTATTGGAGTCTCAACCACAAC
BnAAP8aR	GTATACCTGATAGGCACCTACTAAATGGA
BnAAP8bF	TGAGATCCAGGACACATTGAGATCAAG
BnAAP8bR	CTTCAACGAGTACTCTTTGTTGATGAAATTG

Table S4 Gene family member(s) which could be amplified by the primer pairs used in this work

Primers	Family member(s)	Primers	Family member(s)
BnIPT1a	BnIPT1-2	BnIPT1b	BnIPT1-3
BnIPT2	BnIPT2-1, 2-3	BnIPT3	BnIPT3-1, 3-2
BnIPT5	IPT5-1, 5-2	BnIPT7a	BnIPT7-2
BnIPT7b	BnIPT7-1	BnIPT8	BnIPT8-1, 8-3
BnCKX1a	BnCKX1-1/1-3	BnCKX1b	BnCKX1-3
BnCKX2a	BnCKX2-1, 2-3	BnCKX2b	BnCKX2-2
BnCKX3a	BnCKX3-2, 3-3	BnCKX3b	BnCKX3-1
BnCKX4a	BnCKX4-2	BnCKX4b	BnCKX4-1
BnCKX5a	BnCKX5-1	BnCKX5b	BnCKX5-2
BnCKX6a	BnCKX6-1	BnCKX6b	BnCKX6-2
BnCKX7a	BnCKX7-1	BnCKX7b	BnCKX7-2
BnSUT1a	BnSUT1-1, 1-2, 1-3	BnSUT1b	BnSUT1-1, 1-2
BnSUT2a	BnSUT2-1, 2-2	BnSUT2b	BnSUT2-2
BnSUT3a	BnSUT3-1, 3-2	BnSUT3b	BnSUT3-1
BnSUT4a	BnSUT4-1, 4-2	BnSUT4b	BnSUT4-1, 4-2
BnCWINV1a	BnCWINV1-1	BnCWINV1b	BnCWINV1-2
BnCWINV2a	BnCWINV2-1	BnCWINV2b	BnCWINV2-2
BnCWINV3a	BnCWINV3-2	BnCWINV3b	BnCWINV3-1
BnCWINV4a	BnCWINV4-2	BnCWINV4b	BnCWINV4-1
BnAAP1a	BnAAP1-1, 1-2, 1-3, 1-4	BnAAP1b	BnAAP1-1
BnAAP2a	BnAAP2-2	BnAAP2b	BnAAP2-1
BnAAP3a	BnAAP3-1, 3-2	BnAAP3b	BnAAP3-2
BnAAP4a	BnAAP4-1	BnAAP4b	BnAAP4-1, 4-2, 4-3
BnAAP5a	BnAAP5-2	BnAAP5b	BnAAP5-1
BnAAP6	BnAAP6	BnAAP7a	BnAAP7-1
BnAAP7b	BnAAP7-2	BnAAP8a	BnAAP8-1, 8-3
BnAAP8b	BnAAP8-2		

Table S3 Gene expression profiles during development (Biological replicate 2)

Genes	Expression	L1	L2	L3	F1	F2	F3	P1	P2	P3	P4	P5	P6	P7
BnIPT	Fold change	0.20	23.60	0.86	46.26	1.65	6.15	11.01	91.44	400.19	229.98	82.98	51.24	14.55
1-2	SD	0.43	12.60	0.69	15.39	0.24	1.94	4.27	20.53	119.92	80.91	12.87	9.08	8.61
BnIPT	Fold change	7.22	39.30	2.11	20.06	2.76	3.80	43.06	199.67	232.87	274.77	2.00	2.76	11.37
1-3	SD	2.35	93.27	0.65	6.97	1.86	1.49	24.42	33.34	41.75	57.00	0.94	0.85	4.02
BnIPT	Fold change	2.01	2.14	1.86	1.53	1.57	1.25	1.00	1.15	1.97	2.68	2.28	1.53	1.15
2-1/2-3	SD	0.73	1.22	0.24	0.27	0.60	0.17	0.30	0.38	0.67	1.01	1.09	0.91	0.30
BnIPT	Fold change	8.83	70.75	58.71	3.39	0.73	0.52	87.27	74.28	136.91	278.39	53.92	55.68	18.80
3-1/3-2	SD	2.00	10.74	16.45	1.18	0.25	0.24	12.32	11.94	34.65	56.99	23.85	19.54	12.23
BnIPT	Fold change	8.64	26.14	4.67	3.18	10.37	4.76	17.07	68.37	90.14	69.39	16.47	2.43	3.57
5-1/5-2	SD	10.66	16.16	1.99	1.50	2.28	3.53	9.78	11.33	14.01	17.41	10.38	2.16	2.10
BnIPT	Fold change	4.24	43.03	37.36	1.80	52.13	55.51	77.18	107.49	166.95	118.49	52.18	7.98	1.17
7-1	SD	1.94	16.87	28.39	1.43	11.96	22.70	37.22	26.09	31.93	28.83	6.36	12.59	2.63
BnIPT	Fold change	2.70	9.39	5.84	1.00	1.42	2.03	52.60	108.84	384.67	268.78	140.09	173.71	16.41
8-1/8-3	SD	1.54	3.33	1.75	0.45	1.63	0.94	7.04	23.01	107.05	67.94	35.48	45.05	11.39
BnCKX	Fold change	47.56	229.93	237.63	4.32	2.94	127.05	379.89	1122.93	1038.62	2065.55	145.37	55.13	40.58
1-1/1-3	SD	22.19	59.44	88.87	8.17	5.75	69.19	67.22	387.14	234.07	501.19	40.54	29.52	21.59
BnCKX	Fold change	95.05	96.90	12.78	271.83	83.84	164.86	118.85	1473.67	1024.56	324.47	309.97	242.83	3.90
1-3	SD	19.28	15.02	5.39	83.51	44.96	43.21	58.17	326.29	141.74	80.27	183.29	47.96	5.23
BnCKX	Fold change	1.00	2.43	1.18	6.27	5.33	8.21	6.20	14.80	28.13	38.70	52.67	30.22	12.27
2-1/2-3	SD	0.00	0.91	0.19	5.03	3.12	6.37	7.44	12.73	3.34	15.07	11.15	13.63	5.96
BnCKX	Fold change	1.19	3.37	2.31	18.15	14.62	12.59	7.97	47.81	172.32	162.99	658.38	290.03	51.49
2-2	SD	0.27	3.35	1.85	21.22	12.76	7.97	4.72	24.88	62.26	8.46	78.68	194.45	20.08

BnCKX 3-2/3-3	Fold change	7.69	46.56	10.70	226.85	21.36	5.49	4.21	330.03	540.18	123.20	7.91	4.30	3.37
	SD	1.31	15.32	3.26	87.52	15.40	2.81	2.29	84.82	146.31	65.58	1.72	2.46	2.11
BnCKX 4-1	Fold change	1.00	7.80	2.79	2.79	123.18	474.28	33.96	231.77	1324.46	1315.88	2125.08	60.82	18.33
	SD	0.00	7.72	3.77	1.40	68.65	229.76	9.61	792.78	247.08	120.83	274.22	36.86	9.76
BnCKX 4-2	Fold change	1.00	24.05	2.80	140.09	169.84	10.77	17.00	60.96	124.45	136.57	10.46	1.42	5.13
	SD	0.00	9.56	2.45	25.32	37.54	4.78	4.54	16.17	29.01	30.69	6.12	-0.09	4.86
BnCKX 5-1	Fold change	738.17	275.96	557.97	66.86	65.58	25.75	39.42	164.80	351.45	157.81	103.99	155.99	27.28
	SD	129.12	47.62	173.73	27.14	44.00	28.32	15.54	44.97	76.80	19.73	44.13	37.59	18.72
BnCKX 6-1	Fold change	455.82	820.50	850.79	100.33	35.63	42.16	107.48	342.34	475.89	482.15	216.55	219.33	47.41
	SD	154.34	244.84	132.11	47.46	37.42	13.37	32.53	136.42	30.78	90.95	69.61	51.65	14.10
BnCKX 7-1	Fold change	3.39	12.91	15.28	3.13	3.86	6.80	1.00	5.18	18.96	11.53	6.60	5.06	1.90
	SD	2.71	3.90	7.42	0.94	1.53	8.48	0.20	1.82	13.74	2.24	1.07	2.17	1.08
BnSUT 1-1/1-2/1-3	Fold change	4.69	9.65	7.31	0.38	1.98	3.30	NT	2.58	3.52	2.26	3.76	2.75	2.16
	SD	0.29	0.09	0.29	0.29	0.45	0.68	NT	0.21	0.58	0.11	0.45	1.02	1.23
BnSUT 2-1/2-2	Fold change	4.30	71.25	27.55	1.24	4.44	11.03	NT	6.71	7.92	7.97	16.72	7.37	5.76
	SD	1.99	28.46	3.28	0.12	3.64	5.26	NT	2.13	3.59	1.68	2.92	3.12	2.14
BnSUT 3-1/3-2	Fold change	4.82	107.40	11.97	3.25	4.64	10.26	NT	4.16	3.79	4.28	5.99	1.88	1.65
	SD	1.01	8.97	6.39	0.75	0.79	0.32	NT	0.73	1.34	0.12	1.39	0.46	0.21
BnSUT 4-1/4-2	Fold change	3.47	105.58	28.61	1.19	2.32	7.00	NT	2.29	2.70	2.42	3.92	3.95	3.07
	SD	0.33	35.86	0.55	0.62	1.83	0.16	NT	0.07	0.24	0.22	0.77	0.37	0.82
BnCWINV 1-1	Fold change	7.02	7.28	8.84	3.16	4.26	7.08	NT	24.44	5.83	6.39	4.74	4.40	3.91
	SD	4.00	3.72	2.34	1.26	2.27	3.56	NT	10.21	2.63	0.61	2.33	4.33	4.35
BnCWINV 2-1	Fold change	2.37	5.27	12.03	11.48	91.58	66.13	NT	21.08	17.15	4.95	9.39	3.40	1.27
	SD	1.21	3.90	7.26	9.88	30.48	44.63	NT	9.00	6.21	4.32	6.84	1.34	0.20

BnCWINV	Fold change	0.57	2.25	0.11	2.80	8.98	5.58	NT	6.93	5.74	5.58	5.33	4.37	3.25
2-2	SD	0.42	1.31	0.01	0.96	1.44	1.39	NT	0.99	0.86	0.67	0.86	1.16	0.94
BnCWINV	Fold change	6.27	6.51	6.87	4.71	21.19	20.51	NT	19.42	17.00	12.58	8.00	6.24	1.77
4-1	SD	3.11	3.33	3.64	6.08	10.83	3.37	NT	8.25	12.18	3.28	4.37	1.34	0.68
BnAAP1-<br>1/	Fold change	5.07	8.03	18.52	1.35	6.40	16.39	NT	2.90	3.95	4.16	6.30	3.21	3.42
1-2/1-3/1-4	SD	0.28	3.92	4.46	0.12	0.89	12.80	NT	0.95	0.40	1.18	1.97	1.00	1.23
BnAAP	Fold change	6.90	17.55	8.06	1.10	3.37	6.69	NT	2.79	3.47	3.05	6.20	5.05	4.34
2-1	SD	1.32	2.64	1.46	0.08	0.61	0.71	NT	0.11	0.23	0.62	1.36	1.49	1.23
BnAAP	Fold change	3.82	9.17	13.60	1.26	1.67	2.15	NT	56.01	11.42	51.00	3.17	31.21	8.37
2-2	SD	1.60	4.07	6.75	0.37	0.91	1.29	NT	25.08	5.21	16.37	1.75	15.36	2.23
BnAAP	Fold change	7.71	50.46	5.87	3.18	8.39	17.78	NT	4.15	5.41	3.97	5.72	3.10	2.08
4-1	SD	2.65	6.37	5.66	0.83	1.90	2.40	NT	0.54	1.15	1.50	1.58	0.89	0.75
BnAAP	Fold change	5.76	7.12	11.21	1.83	3.60	1.22	NT	42.14	8.05	35.63	3.54	16.11	4.86
5-1	SD	3.20	2.91	4.03	0.88	2.04	0.30	NT	19.94	4.23	20.01	1.73	6.79	1.80
BnAAP6	Fold change	8.24	11.82	12.11	1.00	1.01	1.35	NT	17.18	5.25	19.90	11.07	13.18	4.56
	SD	0.62	1.40	1.80	0.00	0.00	0.25	NT	2.23	1.08	2.27	2.56	2.25	0.62
BnAAP	Fold change	3.47	5.09	8.56	1.03	1.39	2.37	NT	27.29	6.65	23.60	3.21	10.42	4.56
7-1	SD	0.53	0.70	1.86	0.04	0.19	0.62	NT	6.79	0.17	2.26	0.19	1.09	0.75
BnAAP	Fold change	1.25	3.89	7.46	3.61	1.76	2.50	NT	60.32	18.98	61.30	14.14	35.34	11.91
8-1/8-3	SD	0.26	1.14	2.48	0.50	0.41	1.45	NT	18.72	6.80	29.74	4.01	13.43	3.60

Notes: Relative expression (fold changes) was calculated relative to the lowest expressed sample of the same sample set, and was corrected using the geometric means of reference genes *α-EF1* and *GAPDH*. L1 young expanding leaves, L2 expanded leaves, L3 senescing leaves; F1 flower buds 3-4 mm, F2 flower buds 6-8 mm, F3 open flowers; P1-P7 siliques at different developmental stages, from 15-20 mm to full size of 70-80 mm.
